# Snake Oil or Panacea? How to Misuse AI in Scientific Inquiries of the Human Mind

**DOI:** 10.3390/bs16020219

**Published:** 2026-02-03

**Authors:** René Schlegelmilch, Lenard Dome

**Affiliations:** 1 Department of Psychology, Faculty of Human and Health Sciences, University of Bremen, 28359 Bremen, Germany; 2 Department of Psychiatry and Psychotherapy, Faculty of Medicine, University Tübingen, 72074 Tübingen, Germany; lenarddome@gmail.com; 3 German Center for Mental Health (DZPG), Partner Site Tübingen, 72074 Tübingen, Germany

**Keywords:** large language models, embedding-based regression, out-of-distribution generalization, model adequacy, extrapolation failure

## Abstract

Large language models (LLMs) are increasingly used to predict human behavior from plain-text descriptions of experimental tasks that range from judging disease severity to consequential medical decisions. While these methods promise quick insights without complex psychological theories, we reveal a critical flaw: they often latch onto accidental patterns in the data that seem predictive but collapse when faced with novel experimental conditions. Testing across multiple behavioral studies, we show these models can generate wildly inaccurate predictions, sometimes even reversing true relationships, when applied beyond their training context. Standard validation techniques miss this flaw, creating false confidence in their reliability. We introduce a simple diagnostic tool to spot these failures and urge researchers to prioritize theoretical grounding over statistical convenience. Without this, LLM-driven behavioral predictions risk being scientifically meaningless, despite impressive initial results.

## 1. Introduction

Contemporary artificial neural networks (ANNs), especially large language models (LLMs), are celebrated for their remarkable ability to encode, compress, and generalize diverse inputs, delivering impressive results in natural language processing and beyond. These models act as versatile lenses into complex data, synthesizing rich information into numerical vectors that can be used in statistical regression modeling in a variety of ways (see Zhou et al., 2025). Yet, as enthusiasm for these tools grows, so too must our scientific caution. Whether practically or theoretically motivated, the scientific community must uphold rigorous standards, transparently control for confounds, and remain vigilant of the epistemic limits of these models (Guest & Martin, 2025; Guest & van Rooij, 2025).

This article focuses on a growing class of LLM-based methods called feature extraction or embedding-based regression (Bhatia & Richie, 2024; Hussain et al., 2024; Tang et al., 2024). These frameworks are used to automatically encode text-based information like experimental stimuli and task information to predict human behavior. These approaches leverage tensors, which are compact numerical vectors (or multi-dimensional arrays) of the content in the text-based input. Tensors can be linked to behavior by using their numerical elements as independent regressors in a regression model. For example, in their recent study, Aka and Bhatia (2022) used such models to encode linguistically and semantically rich texts of disease descriptions. Specifically, after converting each disease description into a tensor, they incorporated them into a regression model to predict participants’ severity judgments regarding these diseases. This resulted in an impressive r=0.725 correlation with the true human judgments. At its core, the framework indicates that such models can recognize and quantify the semantic and linguistic regularities woven into these texts. These regularities can correlate with human judgments, allowing embeddings to serve as effective predictors in the absence of a cognitive model.

In this vein, these methods show promise for psychological scaling; however, we argue that without theoretical constraints, they can easily produce “predictions” that are not grounded in psychological reality. As shown later, the predictor–outcome (input–output) links established with these methods often fail to generalize to novel input and behavioral conditions. Despite their promise, we argue that embedding-based regression faces two fundamental limitations when used as scientific models of behavior: (1) susceptibility to spurious correlations hidden in the data and (2) failures of generalization when predictors are evaluated outside of training distributions.

First, if the training data used to estimate regression coefficients contains confounding and incidental variance, as is common in real-world datasets, embedding-based regression may exploit spurious correlations that do not generalize to new behavioral conditions. For example, in the disease-description stimuli used by Aka and Bhatia (2022), linguistic properties can covary with disease severity due to institutional conventions. That is, organizational guidelines may oblige NHS staff to use milder language for severe illnesses to make them sound less worrying. Under this latent-structure account, correlations between embeddings and human severity judgments will not indicate that textual features causally drive perception; rather, both can be the products of the same underlying construct, which limits the interpretability and robustness of the framework.

Besides this fundamental limitation, we focus here on a second, more challenging limitation: training data often fails to cover the full input or output space of psychological variables. Such gaps are rarely evaluated, but we demonstrate that embedding-based predictions can become absurd or even *reverse* true relationships. This raises substantial concerns, especially when using data from scientific experiments, as shown in recent studies (Bhatia & Walasek, 2023; Binz et al., 2025). Experiments often do not record all possible outcomes (a) for ethical or practical reasons, and (b) because scientific studies do not require all data to draw causal inferences. This shortcoming is not detected by the typically applied model validation procedures, known as out-of-sample cross-validation. To show this, we explore an overlooked evaluation strategy: testing model performance on psychological outcomes that fall outside the training ‘distribution’. While similar issues have been raised previously from a more technical background (Hendrycks et al., 2020; Shuttleworth et al., 2024), the broader field needs an accessible perspective that clarifies the practical and scientific limitations of AI (Guest & van Rooij, 2025). We aim to contribute to closing this gap, focusing on embedding-based regression methods in a practical way.

By using publicly available datasets, we illustrate the methodological and practical risks that embedding-based regression methods carry. We demonstrate how training data predicts future, yet-unseen data in different psychological experiments, such as severity judgment (Aka & Bhatia, 2022), neural activation in a repeated-choice task (Binz et al., 2025; Feher da Silva et al., 2023), and binary choice behavior in a decision-by-sampling task (Binz & Schulz, 2023; Hussain et al., 2024; Wilson et al., 2014). We follow the analytical methods previously used and published in flagship journals (Aka & Bhatia, 2022; Bhatia & Walasek, 2023; Binz et al., 2025) and address shortcomings of typically-chosen indices of ‘explained variance’. Consequently, we caution against prematurely asserting genuine psychological insight or theoretical transfer through such methods. For this, we introduce a simple operational measure of model integrity, defined as the stability of predictions for an identical dataset under different training conditions. The index we term the SD score is used as a diagnostic tool to expose methodological limitations, rather than as a novel statistical measure. We also show that prioritizing theoretically meaningful patterns over omnibus variance (Bowers et al., 2023, 2025; Roberts & Pashler, 2000; Wills & Pothos, 2012) pinpoints precise failures of embedding regression to capture key findings—failures that future work can address as a solvable scientific challenge.

In what follows, we first outline the embedding-based regression method as previously applied in psychological research. We then examine the theoretical and practical limits, focusing on model evaluation and the definition of model generalization. A key distinction emerges between *interpolation* (predictions within the training data range) and *extrapolation* (predictions for unseen situations outside that distribution). We show how making this distinction critically affects the validity of the method. The commonly applied random cross-validation technique called ‘out-of-sample’ (OOS) mainly tests interpolation, yielding overly optimistic performance estimates. In contrast, the same models fail when faced with cases that lie outside the training distribution. We argue that true out-of-distribution (OOD) tests are essential for valid assessment of model performance, which we illustrate in the remainder. Our goal is to enable researchers and reviewers, regardless of applied or theoretical aims, to spot the pitfalls and critically scrutinize the usefulness and soundness of embedding-based regression.

## 2. The Embedding-Based Regression Framework

Embedding-based regression connects LLMs’ capacity to convert raw text into numerical vectors (tensors, embeddings) with statistical modeling (Khromov & Singh, 2023; Tang et al., 2024). This approach is appealing because it exploits patterns of word occurrence that capture large portions of human semantic knowledge in scalable vector representations (Bhatia & Richie, 2024). These statistical regularities encode conceptual similarity across many domains. The vector representations, therefore, encode many latent factors present in natural language that cannot be captured in a psychological experiment but can account for substantial variance in human judgments. While being most promising in complex natural scenarios, this approach extends to controlled experiments.^1^ A further appeal of this method is their domain generality: because experimental tasks and stimuli can be described in plain language, virtually any experimental paradigms can be encoded as text and processed by the same pretrained model, without task-specific constraints or requiring full-training of the model (Binz et al., 2025).

The general method proceeds as follows (for a more technical tutorial, see Hussain et al., 2024). Experimental tasks are reformulated as text prompts that reflect the information available to participants before they respond. These make up a prompt. Correspondingly, prompt detail varies widely across studies, from basic experimental trials (Bhatia & Richie, 2024), through text stimuli with extensive context (Aka & Bhatia, 2022), to full experimental histories that include formatting, setup, response options, and choice history (Binz et al., 2025). While prompt engineering merits discussion (P. Liu et al., 2021), we address it briefly in our neural data simulation (Use Case 3) and otherwise focus on the role of the training regime. Next, these prompts are tokenized to create an input sequence for the LLMs. This means that the text–input is broken down into smaller units, such as single words and converted into a numerical representation that models understand.

Next, a pretrained LLM is selected to process the tokenized input sequence. The tokenized input flows through fixed stacks of pretrained connection weights, making up the embedding space of the model. Modern LLMs derive these representations from parameters learned during large-scale pretraining (Peters et al., 2018). Available LLMs also differ in the computational stages between input and final-layer embeddings (our extraction target). A deeper understanding of these encoders is unnecessary here, but interested readers can consult P. Liu et al. (2021), Peters et al. (2018), or Naveed et al. (2024).

**Figure 1:**
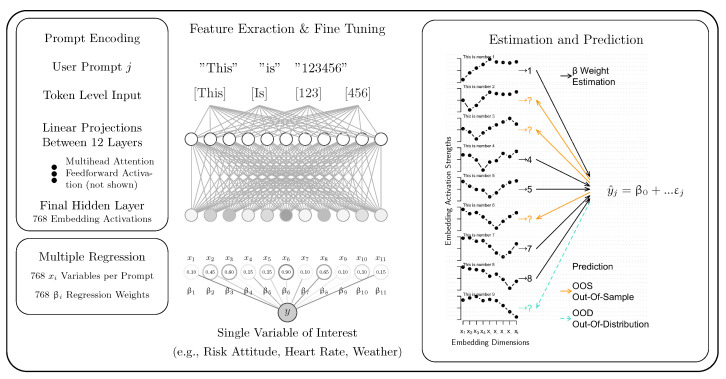
Schematic of feature extraction and cross-validation in the current study. Note: **Feature extraction** outlines how prompts translate into embedding activations, which are then used in multiple regression. **Estimation and prediction** illustrates 10 example prompts like ‘This is number 1’, ‘This is number 2’, …in terms of the strength (y-axis) of the embedding activations (x-axis). Black arrows illustrate examples used to train the regression model (β weight estimation). Orange and turquoise arrows highlight cases that are eligible for out-of-sample (OOS) and out-of-distribution (OOD) tests. See further text.

The third step extracts hidden states from the model’s final layer. These high-dimensional tensors encode the pretrained LLM’s representation of the input sequence, integrating its linguistic and statistical priors acquired during pretraining. Empirical studies demonstrate that late-layer activations exhibit greater discriminability, with the semantic proximity between sequences measured by the distance in embedding space (Bhatia & Walasek, 2023; Bowers et al., 2023). Illustratively, prompts such as “This is number 1” and “This is number 10” produce dissociable 768-dimensional vectors $x_{i}$ in late stages in model architectures such as RoBERTa (Y. Liu et al., 2019). Figure 1 illustrates this process for some example prompts.

In the fourth step, these tensor embeddings serve as input features for a multiple-regression model predicting target behavioral variables, such as human responses. For instance, embeddings from the number prompts could predict participants’ numerical judgments. Researchers typically employ Ridge regression with L2 regularization on high-dimensional tensors used as predictors (coefficients $\beta_{i}$ matching the dimensionality of the tensor), which shrinks weights to mitigate overfitting and multicollinearity (Hastie et al., 2009; James et al., 2013). The linear model then uses the estimated $\beta_{i}$ for generating predictions ($\hat{y}$) without additional theoretical constraints. The $\beta_{i}$ regression coefficients are estimated (calibrated) on a small set of input–outcome pairs (e.g., “This is number 1”∼1; “This is number 10”∼10). This enables predictions for novel inputs, typically called out-of-sample predictions, as those were held out from the training data. A new prompt like “This is number 4”, whose embedding shows partial semantic overlap with training cases (closer to “number 1” than “number 10”, yet distinct from both), yields an intermediate estimate (around 4). Model performance is then evaluated on such held-out cases via cross-validation, typically assessing out-of-sample accuracy (Hastie et al., 2009; Stone, 1974). This means that the collection of prompts is partitioned into a training and test set often by randomly distributing every single prompt into one of the two. Models are trained on the training set, making up the largest chunk of data, and evaluated on the test set. Critical concerns with this approach are addressed in the next section on evaluation and generalizability.^2^

## 3. Generalization, Metrics, and Severe Ordinal Tests

Some embedding-based regression studies claim that their models predict behavioral outcomes on entirely separate datasets based on “out-of-sample” (OOS) evaluation (Bhatia, 2017; Binz et al., 2025; Richie et al., 2019). While such claims attract attention, we argue that they are overly optimistic and can be misleading when OOS involves randomly held-out prompts, which are often unspecified or unjustified despite the existence of other hold-out forms (Hendrycks et al., 2020). Specifically, random OOS primarily tests cases *within* the to-be-predicted behavioral range. In embedding regression, this enables interpolation, where similar prompts predict similar behavior. Critically, as mentioned before, this captures not only semantic but also superficial linguistic features (e.g., prompt length), yielding non-replicable correlations when controlled for in simulation studies (Bowers et al., 2025; Schröder et al., 2025; Xie & Zhu, 2025).^3^ Thus, random OOS can be reinterpreted to reflect only data reliability (consistent outcomes given similar prompts). However, since other cross-validation techniques are seldom applied, we cannot conclude whether this entails theoretical extrapolation of true psychological relationships, or linguistic or other confounds.

This contrasts sharply with empirical psychological research, which does not use all conceivable input variance to explain behavior but instead controls for confounding predictors from the outset (e.g., via experimental manipulations). Methodological control ideally defines what these predictors mean, establishing robust, replicable ordinal phenomena (e.g., group-level differences in cognitive tasks with vs. without time pressure). Hypothesis-driven theorizing traditionally guides experimental designs towards testing for replication, causal influences and assessment of generalization to new experimental contexts (Popper, 1959; Wills & Pothos, 2012; Wilson & Collins, 2019). Embedding-based regression inverts this logic by starting from correlations between prompt-derived representations and behavior and only subsequently asking whether these correlations are psychologically meaningful. This creates a self-referential inference problem: data-derived representations are evaluated by their ability to predict the same or closely related data. From this perspective, predictive success reduces to data predicting data. Our goal is therefore to distinguish theoretical generalization from mere data-to-data interpolation in held-out samples, and we argue that model evaluation should prioritize the nature of hold-out conditions over omnibus measures such as percent explained variance.

Specifically, in psychological science, model *adequacy* assesses whether formal models reproduce theoretically important ordinal trends and heterogeneity in the data (Bowers et al., 2023, 2025; Dome & Wills, 2025; Farrell & Lewandowsky, 2018; Pitt et al., 2006; Roberts & Pashler, 2000; Wills & Pothos, 2012). For embedding regression, the most critical evaluation is whether it reproduces *theoretically constrained ordinal patterns* in human data, as its lack of causal structure makes it prone to mispredicting true relationships when based on superficial prompt similarity. These ordinal patterns are ideally specified a priori as fixed or irreversible hypotheses, rather than inferred post hoc from predictive performance. This forbids models from having “seen” the test data or variations thereof that allow interpolation, which would merely test sensitivity to prompt differences in regression-based analyses (e.g., as a similarity-aware purely descriptive ANOVA). Therefore, we here evaluate ordinal adequacy through model performance under conditions, in which interpolation based on prompt similarity would render false predictions, due to target behavior that falls outside the training distribution (out-of-distribution, OOD) (Hendrycks et al., 2020). Critically, we focus here on the behavioral target variable (actual or hypothetically expected), not on prompt features or contexts, but we briefly discuss the latter in Section Use Case 4.

**Figure 2:**
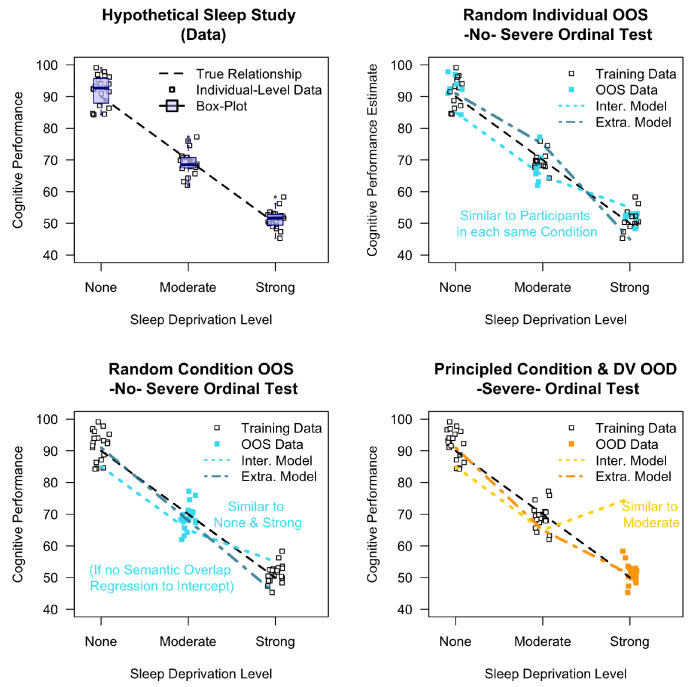
When theory is data: Schematic of (in)severe tests of ordinal success. Hypothetical data of a sleep study, correlating sleep deprivation (x-axis; N = 20 in each condition) with cognitive performance (y-axis; SD = 5 for each mean). Black dashed line indicates the true relation. Inter. Model = Semantic interpolation from previous data. Extra. Model = theoretical 1-factor model (e.g., hours of sleep deprivation predict performance), e.g., simple linear regression. See text for rationale.

Figure 2 illustrates a toy example varying model evaluation setups, where the bottom right panel represents true OOD tests requiring extrapolation. Consider a hypothetical sleep study (top left), in which we assume three sleep deprivation conditions (none, moderate, strong) and measure the cognitive performance of participants (in percent). The smaller dots represent single participant values, of which we assume N = 20 in each condition. We further hypothesize that cognitive performance declines as a function of increased sleep deprivation. Further assume that each participant saw 10 randomly ordered bits of information before being tested on their cognitive performance, such that each participant had an individual ‘prompt’ encoding the stimuli they saw. This is important to note, as previous embedding-regression studies treated these prompt differences as eligible for random OOS hold-outs, as in later sampling-based decision tasks (top right, turquoise symbols; i.e., not by-condition hold-outs Binz et al., 2025; Hussain et al., 2024). Here, the models know the to-be-validated effects of conditions via interpolation between similar prompts, rendering ordinal tests trivially true. The central question is thus, which hold-outs yield stronger tests of whether models capture true theoretical relationships.

For comparison, Figure 2 further contrasts predictions corresponding to the concept of semantic-similarity in an embedding regression (Inter. model = interpolation) against an alternative model (Extra. model = extrapolation). The latter represents our fixed hypothesis captured by a linear regression, with increased sleep deprivation predicting decreased cognitive performance, regardless of the evaluation method. In the bottom left panel, a randomly selected condition (Moderate) is held out and falls in the middle of the behavioral range between None and Strong. That is, Moderate is positioned between semantically neighboring training conditions, which allows for interpolation and results in no differentiation between interpolating and extrapolating models. Note that such semantically-related “in-between” prompts are common in random hold-outs as in our subsequent simulation of Aka and Bhatia (2022) below. Finally, Figure 2 (bottom right) shows a severe test: the consequence of extrapolation failure when relying on semantic interpolation, as the model estimates the behavior based on what is the most similar text input (i.e., the Moderate condition). Embedding-based regression models would never predict such extremes since they are absent from ethical experimental studies. Regardless, such failures are expected in practice.

However, the following simulations consider more complex cases than the three-level sleep deprivation factor. We therefore transition to prompts carrying a recognizable continuum of concepts that we employ later: numbers in prompts such as “This is number 37”. Specifically, assume embedding regressions trained on prompts and behavior for values from 0 to 100 in steps of 10 (e.g., 10, 20, …, 100; see Khromov & Singh, 2023; Tang et al., 2024 on Lipschitz continuity). Models trained on full multiples of ten thus have not seen answers “42” or “150”, making them eligible for hold-out validation. For reference, Table 1 lists six important data hold-out characteristics that could be systematically varied during validation when predicting, for example, number judgments. Note, however, we would expect the same results for predicting number judgments when replacing the numbers inside the prompts (cet. par.) with letters, numerals, or temperature scales, as they preserve local similarity structures (e.g., ‘b’ co-activates patterns of ‘a’ and ‘c’ just as ‘2’ co-activates patterns of ‘1’ and ‘3’, if this concept was pre-trained). That is, not only would the ‘number’ concept correlate with the number judgments, but any concept with an analogous quasi-relational structure.

To clarify key terms in Table 1, we define *in-sample* performance as how well the model fits training data (e.g., correlation between estimated and observed behavior used to fit weights). We use *out-of-sample* (OOS) for prompts absent from training (out-of-prompt-sample), where target behavior is randomly sampled from the full distribution seen during training (in-distribution). Correspondingly, *out-of-distribution* (OOD) refers to cases requiring extrapolation of semantic-behavioral patterns from training data for accuracy, as in the previous examples. Note that ‘distribution’ can also describe prompt features (which we avoid here, specifying those cases explicitly instead). Finally, we use *out-of-domain* for meaningful prompt changes, such as introducing new concepts (Table 1, Cases 5–6) or altering linguistic structure (e.g., shuffling, prompt length).

## 4. Applications and Simulations

We demonstrate key findings through targeted simulations and empirical examples, illuminating the strengths and pitfalls of LLM embedding regression. We focus on comparing OOS and OOD performance of embedding regression in three studies. We begin with a toy example to introduce the general procedure and method illustrated there. We then re-analyze, in this order, a study on the prediction of severity ratings from narrative disease descriptions (Aka & Bhatia, 2022), a study on predicting individual-level neural data using prompt-described stimuli (Binz et al., 2025; Feher da Silva et al., 2023) and a study on predicting the proportion of optimal choices in a binary search horizon task using the prompt-described history of observed choice options (Hussain et al., 2024; Wilson et al., 2014). We emphasize again that we refer to OOD in terms of the (actual or expected) distribution of the behavioral target variable (e.g., cognitive performance in Figure 2), while explicitly outlining what out-of-sample interpolation achieves and what it does not in these studies. Our central question is whether the model predictions adhere to mandated orderings in severe tests or rather self-imposed orderings in data predicting itself instead. In the following, we illustrate instances where the models may not hold up or may even reverse their predictions between OOS and OOD tests.

### 4.1. Simulation Methods

While there are some differences between the methods we employed for each use case due to the nature of the dependent variable (e.g., linear vs. logistic), the overarching framework remains consistent across all use cases. Specifically, we always use the same method to assess model performance under multiple cross-validation regimes designed to separate in-sample, OOS (but in-distribution), and OOD (but in-domain) generalization. This method first generally splits the whole sample into training and test sets. Using the training set, we perform a standard k-fold regularized Ridge regression (R-package glmnet 4.1-10; Friedman et al., 2010), obtaining the weights of all $x_{i}$ embedding variables. We also generate training predictions to assess the fit in terms of correlation (or coefficient of determination in the logistic case). We then pass the held-out test prompt-activated embeddings to the trained model and generate corresponding test-set predictions and evaluate them correspondingly. Below, we describe how we derive our central scoring method based on these estimates. Crucially, the main variation in our design concerns whether prompts were held out in random splits or systematically. For the former, we used 33% random sampling (OOS). For the systematic splits, we used the top or the lowest (or both) of the dependent variable (OOD), ensuring that evaluation requires interpolation and extrapolation in the target space, respectively. We provide further details in each case.

#### SD Score

To quantify ordinal reversals of model predictions, we designed an omnibus score to quantify misalignment between model predictions and data or corrupted model integrity. We call this the SD Score, which we consider alongside other patterns of results for interpretation. We compare the test-set predictions of the model with its predictions on the exact same prompts after exchanging the training set and thereby the model weights. Ideally, the model weights should predict the same behavior in the test set. Specifically, we calculated the ratio between (i) the summed distances of OOD predictions from the data and (ii) the summed distances of in-sample fit from the data. The SD score of 1 indicates model integrity in the cases addressed here (when the fit is high). Accordingly, log(SD Score) should be normally distributed around 0 for consistent models in multiple tests (not only OOS but especially OOD). In contrast, scores larger than 1 reveal that predictions diverge more from the data than the fitted model, signaling breakdowns in extrapolation. Scores smaller than one reflect a worse fit than test performance, which would warrant closer inspection. Here, we expect higher SD Scores in the following simulations, reflecting degraded generalization and unpredictable model behavior in out-of-distribution extrapolation. We apply it throughout because of its ease of interpretability (deviations from 1 are meaningful); it is scale-free and highly sensitive to extrapolation failures of otherwise flexible, well-fitting models. Note, we also report correlation scores in each analysis and Figure, which refer to the correlation between the validation test set and the corresponding predictions. For clarity, in the next section, those values are unrealistically high because we constructed a synthetic data set and the prompts accordingly to highlight the method’s flexibility.

### 4.2. Use Case 1: Interpolation Versus Extrapolation in Synthetic Data

**Figure 3:**
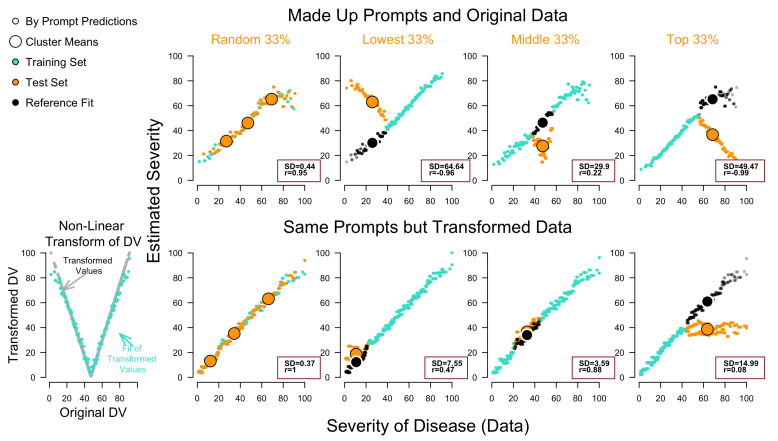
Reanalysis of disease severity judgments (Aka & Bhatia, 2022) using made-up prompts. We used identical prompts in all eight simulations. Columns (Random 33, Lowest, Middle, Top) and corresponding symbol colors indicate which observations/estimates were in the training (turquoise) and the validation test set (orange). Large symbols refer to cluster means, and small symbols to single prompts. Correlations and SD scores refer to the validation test. The black symbols represent reference points of fit for the observations on the validation set estimated for the same data, but in separate runs (fit of test set vs. validation using test set). Diverging estimates indicate failure of extrapolation in out-of-distribution tests in panels Lowest and Top 33% (correlations and/or means = inaccurate), while showing relatively solid interpolation in Random 33% (correlation and means = accurate) and superficial interpolation in Middle 33% (means = somewhat accurate but no correlation).

This is a technical demonstration of how embedding regression behaves when generalizing within the training data domain (interpolation), as opposed to beyond it (extrapolation). To be transparent, we create synthetic data by injecting a made-up relationship between prompts and a behavioral variable. To use a realistic dependent variable, we take the 777 severity ratings from the study of Aka and Bhatia (2022). Originally, the diseases’ descriptions were used as prompts to predict these target ratings, which we address in the next section. In the current toy example, we predict the same data using completely made-up prompts to illustrate how semantic/pattern similarity in embedding regression operates. In principle, the prompt engineering here provides information about the dependent variable itself to the model, similar to assigning prompt labels to the data. For example, prompts like ‘These are low values’ and ‘These are high values’, respectively, could be trained to predict the two values of 10 and 100 (training set), which we here extended to predict behavior in 777 artificial prompts and read out their activated embeddings, keeping this prompt-output mapping constant across all simulated conditions in this section^4^ (for a similar method, see Tang et al., 2024). As holdouts, we used test prompts related to the dependent variable, as shown in Figure 3.

Figure 3 shows (Top Row) the corresponding OOS and OOD tests when synthetically correlating prompts and behavior. As can be seen, when using a random 33% OOS validation, the model exhibits high accuracy and consistency. This is due to interpolation between similar number prompts that preserve the structure of the dependent variable. This structural preservation, however, breaks down in all three OOD cases (Lowest 33%, Middle, and Top 33%). Not only does the correlation between prompts and data entirely reverse in the extreme cases, but when comparing the test-set means (large orange circles) to the fit means (large black circles), one can clearly observe a severe ordinal reversal as well. The boxes in each frame further highlight the corresponding correlations and SD Scores, clearly indicating their sensitivity to the misaligned model predictions. Before applying the same methods to the original study by (Aka & Bhatia, 2022), however, a second, more epistemic failure needs to be acknowledged as well.

Specifically, the bottom row in Figure 3 shows the exact same analysis, using the same prompts, while replacing the dependent variable with its non-linearly transformed pendant (V-shaped transform). As can be seen, the model achieves approximately the same performance in each test. In brief, the epistemic failure of using fit statistics lies in the inability to infer the shape of the relationship in the first place, while the OOD tests reveal the previously described data dependency. We will explore the latter perspective further in the simulation of Feher da Silva et al. (2023) below, as it can be used in procedures like the one here; for example, providing trial indices ‘This is trial 1’, etc., or time horizons, like ‘Three trials to go’, can lead to similar variance binding outcomes.

### 4.3. Use Case 2: Assessment of Disease Severity from Textual Descriptions

**Figure 4:**
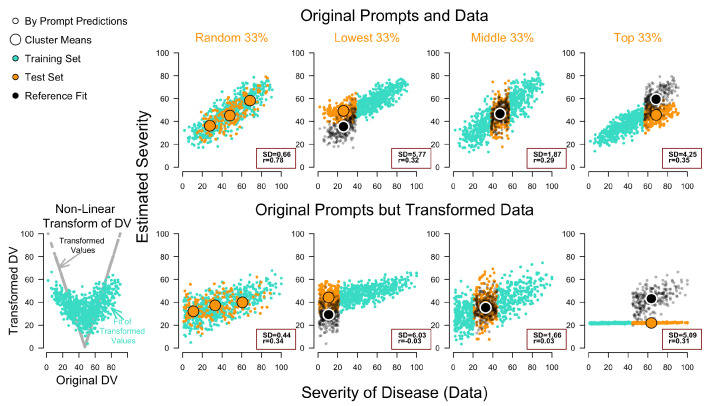
Reanalysis of data by Aka and Bhatia (2022). Columns (Random 33, Lowest, Middle, Top) and corresponding symbol colors indicate which observations/estimates were in the *training* (turquoise) and the *validation* test set (orange). The black symbols represent reference points of fit for the observations on the validation set estimated for the same data, but in separate runs (fit of test set vs. validation using test set). Correlations and SD scores refer to the validation test. Large symbols refer to cluster means, and small symbols to single prompts. The smaller symbols reflect single prompts, and the larger symbols represent cluster means in different ranges of the dependent variable (x-axis; as used in the regression), showing their corresponding model estimates of severity (y-axis). Diverging estimates between predictions (orange) and fit (black), across all three panels, show ordinal reversal of predictions, thus, indicating a severe failure of extrapolation in out-of-distribution tests (Lowest & Top 33%), while showing solid interpolation in Random 33% (correlation + means = accurate) and superficial interpolation in Middle 33% (means = accurate, but no correlation). Note the bottom right predictions appear ‘flat’, reflecting a floor-prediction due to the training data being constrained to the lowest values, i.e., the lower 67% of values in V-shape in the left-hand graph of the transformed dependent variables, which lie between 20 and 30.

We further leverage the dataset introduced by (Aka & Bhatia, 2022), which relates textual descriptions of 777 diseases to human severity ratings for each distinct description (=prompt). Figure 4 applies the same general procedure as described before and as used in the original paper. However, we use a larger OOS set, while basically replicating their result (top Random 33% in Figure 4). We randomly remove 33% of distinct prompts, estimate regression coefficients (or regression weights) on the embedding activations of the remaining prompts (turquoise symbols = fit), and predict the mean human severity ratings for each disease in the held-out test set (orange) by applying their embedding activations. We then evaluated model performance regarding these random out-of-sample (OOS) predictions by correlating them with the corresponding mean severity scores. This method yields a correlation around r=0.7 (depending on the encoder model used). While this result appears impressive and suitable for downstream applications, the stricter OOD tests reveal concerning mis-predictions and outright reversal of the average trends. The SD Scores are correspondingly higher in those cases.

Besides the practical concerns associated with incomplete data regimes, the second row in Figure 4 shows a similar but weaker trend, as in our toy example. We performed this simulation just as for the made-up data in the previous section (Non-Linear Transform of DV). As can be seen, despite this severe transformation, the prompts still capture a ‘substantial’ or ‘moderate’ labeled correlation in OOS of r=0.38, but again an ordinal reversal in OOD, highlighting that the method does not readily distinguish between linear and quadratic relations. In the next section, we demonstrate how this flexibility can also track auto-correlations in neural data simply by providing the trial number to the model. These cases highlight that the ceiling of explained variance in some applications, depending on the semantic structure within the prompt, can easily approach 100%, but which can be entirely meaningless, and comes at the cost of scientific reduction or theoretical insight.

### 4.4. Use Case 3: Individual Neural Correlations

In this section, we shift our focus from applied cases to illustrate the methodological limitations within a highly controlled laboratory setting. We examine the embedding regression used to predict trial-wise (150 trials) brain activation for individual participants, who were scanned in an fMRI while completing a repeated-choice paradigm Feher da Silva et al. (2023). Binz et al. (2025) described an analysis of this data, in which activity within each neural region was aggregated and regressed onto CENTAUR’s representations, following an embedding-regression method. They note that this analysis benefited from using high-dimensional embeddings, outperforming cognitive models. To further explore this claim, we apply a comparable approach using the same neural dataset from Feher da Silva et al. (2023), but with a much simpler RoBERTa-based encoding method (computable in approximately 2 min) and an alternative prompting method.

**Figure 5:**
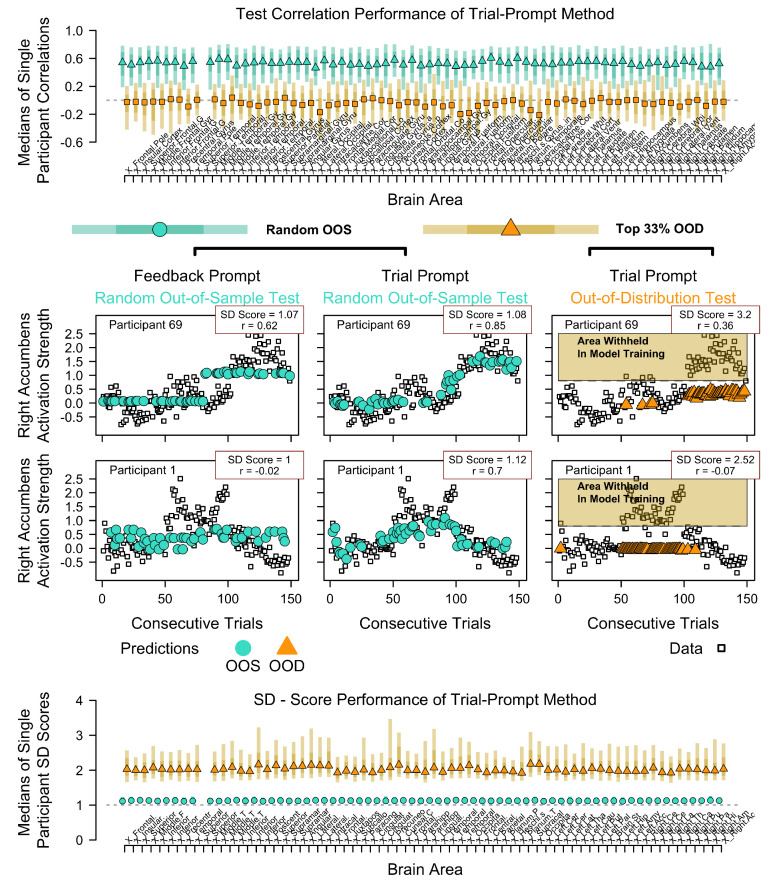
Reanalysis of neural correlations. Data by Feher da Silva et al. (2023). Analysis similar to the analyses in Binz et al. (2025). Trial prompt method refers to prompting each trial *t* as “This is Trial *t*” (with t=1,2,3…). Feedback method refers to prompts only indicating the rewarded choice option. (**Top**) Medians (y-axis) of individual correlations in the respective brain area (x-axis) between trial-wise activity and the estimates of the embedding regression (dashed horizontal line = 0 for reference). Out-of-sample (OOS) and out-of-distribution (OOD) cross-validation is indicated by colors and symbol shapes. (**Middle**) Two example participants highlight two corresponding prompting methods and the two cross-validation comparisons. Small squares depict single-trial data, colored symbols depict predictions for withheld test data. OOS predictions via ‘trial prompt method’ (turquoise, mid column) are due to model interpolation of auto-correlation patterns (similar trials ∼ similar blood flow/activity). Orange symbols in right-hand panels reflect OOD predictions, when these were the 33% highest values withheld from training, showing a total collapse of model performance (no extrapolation if more extreme brain activity was missing from the training set). (**Bottom**) SD scores for the trial-prompt model in the two evaluation methods (dashed horizontal line = 1 for reference), highlighting severe instability in predictions, and lack of model integrity, as also seen in the individual examples. Color shading in bars indicate 50% and 80% range of individual correlations and SD scores.

Figure 5 illustrates the main results when assessing correlations (top) and SD Scores (bottom) in OOD and OOS test sets, when the model was trained on prompts containing information about the trial number (e.g., ‘This is trial 1’, etc.), labeled Trial-Prompt Method. As can be seen, this method excelled in capturing neural trial-wise variance when performing each regression individually and with random OOS tests. However, it failed in OOD extrapolation tests, yielding zero correlations and high SD Scores. To make it clear why the OOS Trial-Prompt Method captures variance, the middle group of panels in Figure 5 shows two example participants (rows). For comparison, the left middle panel shows the model predictions when only presenting prompts that contain the choice options and the feedback for a given trial (Feedback Prompt). The two right-hand panels show the Trial-Prompt Method for the OOS and OOD methods, respectively. As can be seen, providing semantic prompt structure in terms of ascending prompts is enough to achieve median correlations of r∼0.6 by binding auto-correlation patterns that would otherwise likely be interpreted as noise. The OOD method further demonstrates that performance is not due to the richness of representations but, as before, to the richness and semantic structure of the prompt input, which should be chosen wisely before conducting such variance analyses (e.g., in a factorial model comparison), as it otherwise can give a false impression of explained/explainable variance (e.g., in this case, the trial prompt method ‘explains’ nothing more beyond the DV time-series being auto-correlated).

### 4.5. Use Case 4: Choice Variability Modulates Explore–Exploitation Tradeoff

In the final simulation, we explore the use of embeddings to predict binary choice behavior in a decision-making task, examining the ordinal relationships between theoretically motivated experimental conditions. We show that the method, when applied to this setting, can lead to spurious predictions arising from generalizations analogous to the previous cases. To do so, we use the publicly available dataset of Wilson et al. (2014) and follow a well-documented tutorial by Hussain et al. (2024), reflecting a method described previously in Figure 1 (right panel; OOS), which we compare with OOD tests.

**Figure 6:**
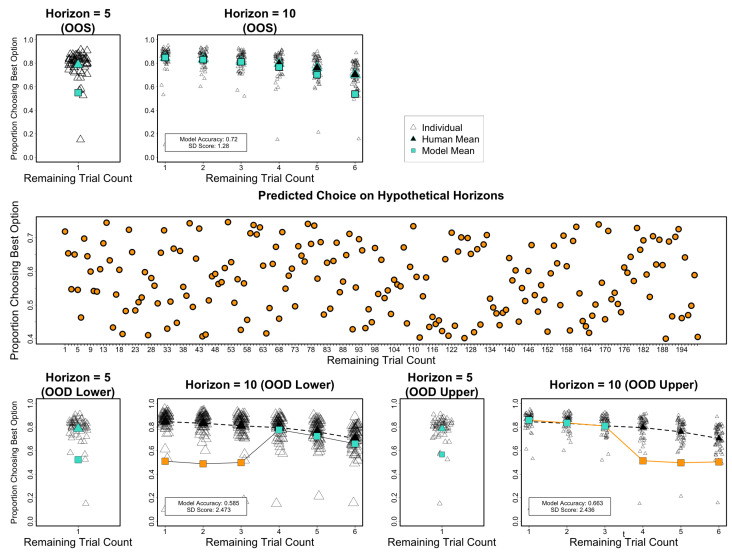
Simulation of Horizon Task by (Wilson et al., 2014). Human choice proportion (y-axes) of the best option and model predictions based on random out-of-sample (**Top** and **bottom row**) tests in both horizon conditions (Horizon = 5 vs. 10, respectively, with 1 and 6 remaining trials [x-axis] after sampling of both options). Model accuracy and SD scores refer to validation test. (**Middle row**) Remaining trials (prompted, y-axis) and predictions of proportion best choices (x-axis), representing ‘out-of-distribution’ due expected increase in exploiting the best choice with experience, but not predicted by the OOS model from top row. (**Bottom row**) Performance when using out-of-sample and out-of-domain prompts (OOD, prompts). The training and test sets differ as indicated by orange and turquoise symbols, representing the model predictions, when holding out cases of remaining trials 1–3 or 4–6 (held-out region = orange; training set = turquoise). Large dark triangles/dashed line reflects data means.

Wilson et al. (2014) developed a multi-armed bandit task in which each arm incorporates prior information regarding the expected reward of that arm. This prior information is not always reliable and introduces uncertainty in decision-making. The primary manipulation in their study involved two conditions: in one condition (Horizon 6), participants were allowed to make one to six consecutive choices following the display of prior information, while in the other condition (Horizon 1), they were only allowed one. The conditions differed in the number of choices allowed—the choice horizon. The central finding of their paper is that exploration in the first trial was more prevalent when there were still six trials (Horizon 10) than when there was just one remaining trial (Horizon 5). Furthermore, in the Horizon 10 condition, exploitation increased with fewer remaining trials, as illustrated in Figure 6. Simply put, participants tended to take less risk and exploit more when the choice horizon was small, while they engaged in more risk-taking and exploration when the choice horizon was larger, but then switched to exploitation. Note that there are more subtle factorial interactions addressed in that study, but this part of the pattern suffices to illustrate the role of testing ordinal trends both within and between conditions.

Both Hussain et al. (2024) and Binz et al. (2025) included this phenomenon in their respective model benchmarks, but neither of them demonstrated the extent to which this ordinal difference is captured. Instead, they reported an index of explained variance (e.g., model performance divided by the performance of a guessing model). While, Binz and Schulz (2023) demonstrated that their early version of the CENTAUR model could capture ordinal differences, but recent investigations revealed that the model’s predictive performance was influenced by several artifacts, including the methods used to translate the experiment into prompts and input them into the model (Xie & Zhu, 2025) and the extent to which it can exhibit behavior that diverges from that of humans (Bowers et al., 2025; Gao et al., 2025). In this vein, note that we followed the method by Hussain et al. (2024) in which the prompts included information about how many trials were left to clearly highlight the issue. That is, providing an index of how many trials are yet to come is similar to our previous prompting methods (Feedback vs. Trial–Prompt Method in the previous section), which also affected the OOS cases.

Briefly, we compiled prompts to include information about a single game (a single choice). Following a brief introduction to the problem, which includes the prior samples for each bandit in a bullet-point list and the remaining trials for making choices, the prompt asks the LLM to choose between the two bandits in the current trial. These prompts were then input to DistilBERT (Sanh et al., 2020), from which we extracted the final-layer embedding to be passed to the logistic Ridge regression in our method.

The top row in Figure 6 (top two left panels) shows that the model failed to reproduce the ordinal differences between the horizon conditions and strongly overestimated them, despite achieving an otherwise convincing accuracy score of 0.717 in this random OOS test. This highlights the risk of over-relying on predictive accuracy, which may lead to a preference for models that do not adequately represent ordinal empirical trends. The bottom row in Figure 5 further shows the effect of systematically withholding prompts in training, indicating how many trials are left (1–3 or 4–6) in the Horizon 10 condition. Note, this also means that trials of different overall lengths were used in training. That is, both factors might contribute to the apparent drop in performance on the held-out test sets (orange).

Finally, the middle row in Figure 5 shows a quasi-OOD test. Specifically, it is typically expected that, as also observed by Wilson et al. (2014), the exploitation of the best option in this task should increase with ongoing trials (e.g., when going up to 200 trials; Feher da Silva et al., 2017; Shanks et al., 2002). Thus, based on previous research, we expect even more extreme levels of Best Choice proportions when continuing the trials. We reused the model parameters estimated in the top row of Figure 6. The middle panel shows the corresponding prediction, or rather the apparent lack of the just-described plausible expectation (trial prompt on the x-axis, predicted choice proportion on the y-axis). Overall, the simulations here demonstrate that the interpolation method in embedding regression achieves moderate to good accuracy (approximately 0.7) in OOS tests, even when failing to replicate theoretically relevant ordinal behavioral trends. The out-of-distribution (OOD) tests further demonstrated a worrying failure to generalize the model’s predictions when factorial information (numbers associated with remaining trials) is removed from the prompts in the training set. This suggests that the model, when evaluated as such, may not be scientifically meaningful, which we discuss more broadly in the following.

## 5. Discussion

Our simulations consistently demonstrate that embedding regression typically achieves robust *in-distribution* predictive accuracy (e.g., random out-of-sample tests) but catastrophically fails under *out-of-distribution* (OOD) extrapolation or out-of-domain (prompt variation), up to reversing ordinal trends and yielding unstable model behavior between prompt environments (SD Scores > 1). This reveals a critical epistemic flaw: the method conflates semantic interpolation within training data manifolds with genuine theoretical generalization, mistaking pattern recognition for scientific explanation. We discuss some implications here.

First, the core scientific limitation of embedding-based regression lies in the distinction between descriptive utility and explanatory validity. The method effectively binds variance by exploiting prompt-derived representations and outcomes, excelling as a *similarity-aware ANOVA*. However, it remains agnostic to the causal mechanism that generates those associations. As a result, even strong predictive performance may reflect trivial or artefactual structural regularities in the data, such as autocorrelation induced by prompt design (e.g., this is trial $t^{\prime \prime }$ capturing auto-correlations in neural data, as shown in Figure 5). Embedding regression summarizes the variance structure in behavioral data, but summarizing all conceivable variance is neither equivalent nor even close to explaining the processes that generate behavior.

In this vein, one could summarize the implication in B. F. Skinner’s words: “It is possible that any example of postulational method in the empirical sciences may be interpreted in the same way and that ‘predicting’ a fact from a set of assumptions is never intended to mean more than describing it. But if this is the case, the pretense of deduction should be abandoned, at least by those who are interested in a descriptive science of thought and who wish, therefore, to see the number of thought processes reduced to a minimum.” (Skinner, 1942, p. 503).^5^

While embedding-based regression could preserve predictive performance in methods other than discussed here, its descriptive success in OOS cases obscures a critical failure revealed under OOD conditions. Even if one would be able to rule out confounds driving these correlations, models trained on disease severity ratings (1–100) can reverse trends for OOD cases, when model training uses aggregated participant means and thus a restricted behavioral range. Such OOD failures violate basic benchmarks of scientific adequacy, known as severe ordinal testing (Bowers et al., 2023; Roberts & Pashler, 2000; Wills & Pothos, 2012). This issue can become acute when extending predictions to individual differences and minority cases. This makes OOD validation a necessary corrective. At minimum, scientific researchers using these methods need to be aware of these failures, as they show that high explained variance reflects statistical convenience rather than explanatory power.

This critique does not preclude theoretically principled integrations of embeddings with cognitive models. Note that there are several interesting and promising approaches that combine embeddings with meaningful cognitive models. For example, deep neural networks have been repeatedly used to extend existing categorization models (Luo et al., 2025; Singh et al., 2020; Tartaglini et al., 2021) or to contrast prototype and exemplar models of categorization using naturalistic stimuli (Battleday et al., 2020; Nosofsky et al., 2022). In non-psychological settings, LLMs have provided rich, grounded hypothesis spaces through interpretable numerical distributions conditioned on text (Requeima et al., 2024). The critical distinction lies in whether embeddings *constrain* theoretical models or whether models become subservient to data-driven interpolation and flexibly adapt to any desired prompt-data structure. We therefore advocate adopting stricter criteria for embedding-based regression to better align with scientific standards. We mandate three guardrails: (1) *require OOD testing* for all generalization claims, (2) *reject explanatory interpretations* of in-distribution explained variance even before treating results strictly as descriptive, and (3) *preserve theory-driven hypothesis testing* where predictions are specified *before* data observation. Due to the lack of complete behavioral distributions and also for ethical reasons, the OOD cases are concerning, and future research needs to show how to further secure the validity of the method in a “similarity-aware ANOVA” under stricter theoretical design.

## 6. Conclusions

We conclude with an urgent message that embedding regression with LLM embeddings, while powerful for data compression and descriptive scaling, currently lacks the theoretical grounding to serve as standalone tools for hypothesis testing or causal discovery in psychology. The scientific community must uphold rigorous standards, transparently control for confounds, and remain vigilant of the epistemic limits of these models.

## Figures and Tables

**Table 1:** Generalization types in prompt-based regression tasks using LLMs. After training a model on prompt-target pairs of multiples of 10 between 0 and 100, all rows except Cases 1–2 reflect generalization challenges, often linked to prompt sensitivity. Note, stronger Embedding Shift implies worse model integrity (mispredictions).

	Test Prompt	Sample Type	Expected Behavior	Embedding Shift
1	“This is number 37” (seen in training)	In-sample	Perfect prediction; reproduces training example	None—seen input
2	“This is number 42” (unseen in 1–100)	Out-of-sample In-distribution	Accurate; generalizes within seen structure	Very low
3	“This is number 150”	Out-of-distribution	Model may fail or invert trend	Moderate–high—numeric embeddings misaligned beyond range
4	“This is NOT disease 42”	Out-of-sample In-distribution Mild out-of-domain	Degraded or biased output; changed prompt alters reasoning	Moderate—semantic framing shifts attention
5	“Xylozyme rank 42 is peak flux”	Out-of-sample In-distribution Strong out-of-domain	Output unpredictable; input is nonsensical or adversarial	Very high—embedding distant from training manifold
6	“Warning: number 150 detected”	Full out-of-distribution (prompt domain and dependent variable new)	Highly unstable or default-biased prediction	High—both prompt and value unfamiliar

## Data Availability

No new data were created or analyzed in this study. All analysis code and data is shared on OSF under https://osf.io/vte78.

## Notes

1 The broadest claim for use-cases in experimental studies is detecting unacknowledged sources of explained variance (e.g., Binz et al., 2025), ostensibly yielding “explainable” variance for theory. Yet this term, as we argue later, is ill-defined: it conflates confounds with meaningful signals and correlation with causation, as elaborated below.

2 Constant prompt prefixes may enhance encoding of varying elements, but using only unique information often improves fit here, suggesting extraneous text can dilute signal (beyond this review’s scope).

3 These outcomes are in line with a broader range of critiques on theoretical interpretability (Cummins, 2025; Guest & Martin, 2023, 2025; Orr et al., 2025).

4 We prefer not to disclose the exact prompt engineering because it would be too easy to conduct severe misuse of it, which we do not want to replicate.

5 In this quote, Skinner originally refers to earlier studies addressing binary choices under uncertainty (then still called ‘guessing’) via the concept of telepathy, instead of a principled set of explicitly stated psychological hypotheses.
